# Tailoring Treatment in Localized Prostate Cancer: Comparative Effectiveness of HIFU, Cryoablation, and Robot-Assisted Radical Prostatectomy at 2-Year Follow-Up: Insights from Prospective Institutional Cohort

**DOI:** 10.3390/cancers17172762

**Published:** 2025-08-25

**Authors:** Umberto Anceschi, Francesco Prata, Rocco Simone Flammia, Andrea Iannuzzi, Eugenio Bologna, Aldo Brassetti, Leslie Claire Licari, Flavia Proietti, Alfredo Maria Bove, Leonardo Misuraca, Gabriele Tuderti, Mariaconsiglia Ferriero, Riccardo Mastroianni, Rocco Papalia, Franco Lugnani, Aldo Di Blasi, Salvatore Guaglianone, Costantino Leonardo, Giuseppe Simone

**Affiliations:** 1 Uro-Oncology Program, IRCCS “Regina Elena” National Cancer Institute, Via Elio Chianesi 53, 00144 Rome, Italy; umberto.anceschi@gmail.com (U.A.); roccosimone92@gmail.com (R.S.F.); eugenio.bologna@ifo.it (E.B.); aldo.brassetti@gmail.com (A.B.); leslieclaire.licari@ifo.it (L.C.L.); flavia.proietti@ifo.it (F.P.); alfredo.bove@ifo.it (A.M.B.); leonardo.misuraca@ifo.it (L.M.); gabriele.tuderti@gmail.com (G.T.); marilia.ferriero@gmail.com (M.F.); riccardomastroianniroma@gmail.com (R.M.); salvatore.guaglianone@ifo.it (S.G.); puldet@gmail.com (G.S.); 2 Department of Urology, Fondazione Policlinico Universitario Campus Bio-Medico, Via Álvaro del Portillo, 200, 00128 Rome, Italy; rocco.papalia@policlinicocampus.it; 3 Research Unit of Urology, Department of Medicine and Surgery, Università Campus Bio-Medico di Roma, Via Álvaro del Portillo, 21, 00128 Rome, Italy; andrea.iannuzzi@unicampus.it; 4 Department of Radiology, Tivoli Hospital, Via Parrozzani 3, 00019 Tivoli, Italy; aldo.diblasi@aslroma5.it

**Keywords:** prostate cancer, RARP, PGC, HIFU

## Abstract

**Background:** In the evolving landscape of localized prostate cancer management, focal therapies such as high-intensity focused ultrasound (HIFU) and prostate gland cryoablation (PGC) have emerged as organ-sparing alternatives for patients with low- to intermediate-risk disease. While these strategies aim to preserve functional outcomes, comparative data against robot-assisted radical prostatectomy (RARP) remain scarce and heterogeneous. **Methods:** We conducted a prospective, single-center study evaluating oncologic and functional outcomes in patients with organ-confined prostate cancer (Grade Group ≤ 2) treated with HIFU (*n* = 49), PGC (*n* = 114), or RARP (*n* = 109). Outcomes were assessed using standardized definitions at a median follow-up of 22 months. Treatment failure was defined according to EAU guidelines, and Kaplan–Meier analysis was applied to time-to-event outcomes. **Results:** Focal therapy patients were older, more comorbid, and had lower baseline erectile function (each *p* < 0.001). RARP was associated with the longest operative time but yielded the lowest complication rate (2.75% vs. 20.4% for HIFU and 31.5% for PGC; *p* < 0.001). Catheter-related morbidity was disproportionately higher in the PGC group. RARP conferred a longer time to treatment failure (*p* < 0.001), although continence and potency recovery at follow-up were comparable across groups. Notably, erectile function returned earlier among HIFU patients. **Conclusions:** While focal therapies offer promising early functional results with minimal perioperative risk, they are associated with earlier treatment failure and higher catheter-related morbidity, particularly after cryoablation. These findings underscore the need for individualized treatment strategies guided by standardized, comparative outcome frameworks.

## Simple Summary

Prostate cancer confined to the gland can be treated with surgery or newer, less invasive techniques. This study compared three options in 272 patients with low- or intermediate-risk cancer: robotic prostate removal, cryoablation, and treatment with focused ultrasound (HIFU). After two years, we found that ultrasounds led to a faster early recovery of sexual function, but it was also linked to a higher risk of cancer coming back. Cryoablation had more complications, mainly due to the longer catheter use after the procedure. Robotic surgery showed better long-term recovery of sexual function, with similar cancer control compared to the other treatments. These results suggest that each treatment has specific advantages and limitations. Understanding these differences can help patients and doctors choose the most suitable approach based on individual needs, preferences, and health status.

## 1. Introduction

Minimally invasive ablative approaches—namely high-intensity focused ultrasound (HIFU) and prostate gland cryoablation (PGC)—have gained a foothold in the therapeutic armamentarium for organ-confined prostate adenocarcinoma, particularly in patients with low- to intermediate-risk disease [1]. These focal strategies aim to reconcile oncologic efficacy with preservation of quality of life, offering reduced perioperative morbidity, shorter recovery times, and the inherent advantage of re-treatability [2,3].

However, comparative data between focal therapies and robot-assisted radical prostatectomy (RARP)—the current gold standard for whole-gland treatment—remain scarce and methodologically fragmented. Most available studies explore isolated endpoints such as surgical morbidity, oncologic control, or functional outcomes, often neglecting comprehensive, head-to-head evaluations across standardized clinical domains. Importantly, despite intrinsic differences in defining oncologic success between extirpative and ablative modalities, functional outcomes—namely urinary continence and erectile function—remain universally applicable and clinically meaningful [4,5,6,7,8,9].

In this context, we performed a prospective, single-center comparative analysis of HIFU, PGC, and RARP in patients with organ-confined, low- to intermediate-risk prostate cancer. By applying harmonized definitions and outcome metrics, we sought to provide an integrated assessment of perioperative morbidity, oncologic durability, and functional recovery at early follow-up, thus addressing a longstanding gap in the comparative effectiveness literature.

## 2. Materials and Methods

### 2.1. Study Design and Patient Cohort

This prospective, board-approved comparative effectiveness study was conducted at a single tertiary referral center (IRCCS Regina Elena National Cancer Institute, Rome, Italy). Between January 2019 and October 2024, we prospectively identified patients with organ-confined prostate adenocarcinoma and prognostic Grade Group (GG) ≤ 2 from our institutional prostate cancer dataset.

All patients underwent a uniform diagnostic work-up including multiparametric magnetic resonance imaging (mpMRI) and either choline or prostate-specific membrane antigen (PSMA) positron emission tomography-computed tomography (PET-CT) for staging [10,11,12]. Patients with radiologic suspicion of nodal or distant metastases were excluded. In order to maintain oncologic homogeneity across groups, we also excluded patients in the RARP cohort who underwent pelvic lymph node dissection. Treatment allocation was based on multidisciplinary counselling and informed patient preference.

All participants provided written informed consent prior to enrolment. The study protocol was reviewed and approved by the institutional ethics committee of IRCCS Regina Elena. Although no formal approval number was assigned due to the internal registry-based nature of the investigation, full ethical clearance was granted in accordance with the Declaration of Helsinki and institutional governance policies at the time of study initiation.

A total of 272 patients met the inclusion criteria and were stratified into three treatment groups: partial high-intensity focused ultrasound (HIFU; *n* = 49), prostate gland cryoablation (PGC; *n* = 114), and RARP (*n* = 109). All procedures were performed by experienced urologic surgeons at our institution.

### 2.2. Baseline and Preoperative Parameters

Baseline variables included age, body mass index (BMI), American Society of Anesthesiologists (ASA) score (I–IV), preoperative prostate-specific antigen (PSA), International Index of Erectile Function-5 (IIEF-5) score (range 1–25), and Prostate Imaging Reporting and Data System (PIRADS) score (2–5). Prostate volume was calculated on multiparametric MRI (mpMRI) using the ellipsoid formula. Biopsy-related variables included total number of cores, number and percentage of positive cores, PIRADS area size (mm), and Grade Group (GG1–3). Clinical T stage and year of treatment were also recorded.

### 2.3. Treatment and Perioperative Variables

All procedures were performed using standardized, commercially available platforms: the Da Vinci Xi® surgical system (Intuitive Surgical, Inc., Sunnyvale, CA, USA) for RARP; the Focal One® system (EDAP TMS S.A., Vaulx-en-Velin, France) for HIFU; and the Cryocare CS Surgical System (Endocare, Inc., Austin, TX, USA; owner/operator: Varian Medical Systems, Palo Alto, CA, USA) for PGC. Perioperative parameters included operative time (minutes), hospital stay (days), and duration of catheterization (days). Nerve-sparing intent during RARP was categorized as bilateral (intrafascial), unilateral (interfascial), or absent (extrafascial). Complications were recorded and graded according to the Clavien–Dindo classification.

In patients undergoing HIFU or PGC, treatment planning followed standardized institutional protocols integrating preoperative mpMRI, targeted biopsy mapping, and multidisciplinary evaluation to define candidacy and the precise ablation template. Eligibility criteria for focal therapy included organ-confined, biopsy-confirmed prostate cancer (Grade Group 1– 2), absence of radiologic extracapsular extension, and no evidence of nodal or distant metastases on baseline staging. Curative intent was explicitly maintained across all procedures. No known cancerous lesions—including Grade Group 1—were deliberately left untreated.

Partial-gland ablation was applied in the majority of HIFU and PGC cases, typically encompassing the index lesion and, when feasible, additional foci located within the same hemisphere. Whole-gland ablation was reserved for bilateral involvement or extensive multifocal disease not amenable to focal targeting. The treatment field was individually tailored based on lesion location, prostate anatomy, and functional considerations. Percentages and ablation patterns are detailed in Table 1.

Post-treatment surveillance included serial PSA measurements, mpMRI at regular intervals, and PSMA- or choline-based PET/CT imaging in the event of biochemical or clinical suspicion. Recurrence after focal therapy was defined as radiologic and/or histologic evidence of clinically significant prostate cancer (Grade Group ≥ 2) following initial treatment. Prostate biopsy was repeated when imaging was inconclusive (e.g., PIRADS 3), while direct retreatment was occasionally pursued in patients with PIRADS 4–5 lesions with concordant PET findings and rising PSA. In all included recurrence cases, histologic confirmation was obtained prior to reclassification or salvage therapy.

Recurrence sites were anatomically categorized as in-field (within the previous ablation zone), out-of-field (elsewhere within the prostate), or distant (extraprostatic). This classification was established through retrospective imaging and procedural map correlation. All cases of systemic progression were radiologically confirmed.

### 2.4. Oncologic Outcomes

Follow-up duration was recorded in months (median, interquartile range (IQR)). PSA surveillance was conducted using treatment-specific protocols: RARP patients were monitored every 3 months for the first year, then every 6 months until year five, and annually thereafter. For patients treated with HIFU or PGC, PSA was assessed every 3–4 months during the first year and biannually thereafter. Treatment failure was defined according to the European Association of Urology (EAU) guidelines: in the RARP group, as a confirmed PSA ≥ 0.2 ng/mL; in the focal therapy groups, as PSA rise with radiological or histological confirmation of recurrence or persistence; or the need for salvage intervention. The onset of metastatic disease at any time was also considered treatment failure in all groups. Time-to-failure was assessed using Kaplan–Meier analysis.

### 2.5. Functional Outcomes

Continence recovery was defined as complete dryness or use of one safety pad. Erectile function recovery was stratified as follows: spontaneous erections, response to phosphodiesterase type 5 (PDE5) inhibitors, use of prostaglandin E1 (PGE-1), or complete erectile dysfunction. Functional outcomes were evaluated at last available follow-up.

### 2.6. Statistical Analysis

Continuous variables were expressed as medians with IQR and compared using one-way analysis of variance (ANOVA) or Kruskal–Wallis test, as appropriate. Categorical variables were compared using the chi-square or Fisher’s exact test. Kaplan–Meier curves were used for time-to-event outcomes, with log-rank test for intergroup comparisons. A two-sided *p*-value < 0.05 was considered statistically significant. All analyses were performed using SPSS Statistics version 28.0 (IBM Corp., Armonk, NY, USA).

## 3. Results

### 3.1. Baseline and Perioperative Characteristics

At baseline, patients treated with RARP were significantly younger than those undergoing either HIFU or PGC, with a median age of 63 years (interquartile range (IQR) 59–67) compared to 74 years in both focal therapy groups (each *p* < 0.001 in Table 1).

Among patients undergoing focal therapy, partial-gland ablation was performed in 83.7% of HIFU cases and 79.8% of PGC cases. Whole-gland ablation was reserved for patients with bilateral involvement or extensive multifocal disease not amenable to focal targeting. Detailed ablation patterns are summarized in Table 1.

RARP patients also exhibited more favorable preoperative characteristics, including lower ASA scores (90.8% classified as ASA I–II, vs. 81.6% for HIFU and 57% for PGC; each *p* < 0.001), and higher erectile function at baseline, as measured by the IIEF-5 (median score 21 (IQR 18–22) vs. 13.5 (IQR 4.5–21) in HIFU and 11 (IQR 3–19) in PGC; each *p* < 0.001). No significant differences were observed among the three groups regarding body mass index (BMI), preoperative prostate-specific antigen (PSA) levels, prostate volume, number of biopsy cores, percentage of positive cores, or PIRADS distribution (each *p* > 0.1).

Intra- and perioperative metrics varied considerably across treatment arms. Operative time was longest for RARP (median of 120 min with an IQR of 106–141), significantly exceeding durations recorded for PGC (55 min with an IQR of 40–65) and HIFU (45 min with an IQR of 35–62) (*p* < 0.001 as shown in Table 2). The length of hospital stay was shortest after RARP (median of 2 days with an IQR of 2–2), followed by PGC (2 days with an IQR of 2–3) and HIFU (3 days with an IQR of 3–3) (*p* < 0.001). Catheterization time was significantly prolonged in the PGC group (median 15 days), compared to 7 days in both the HIFU and RARP cohorts (*p* < 0.001).

### 3.2. Complications

The overall complication rate was highest in the PGC group (31.5%), followed by HIFU (20.4%), and was lowest after RARP (2.75%) (each *p* < 0.001 in Table 2). In focal therapy patients, most complications were catheter-related and included orchitis, febrile episodes, hematuria, and acute urinary retention. Specifically, PGC was associated with 17 cases of orchitis, 8 episodes of fever, 5 cases of hematuria, and 5 instances of acute retention. The HIFU group experienced five episodes of orchitis and three of hematuria. Severe complications (Clavien–Dindo grade ≥ 3) were rare in all groups and did not differ significantly (each *p* > 0.4). No catheter-related adverse events were reported in the RARP cohort.

### 3.3. Oncologic Outcomes

**Figure 1:**
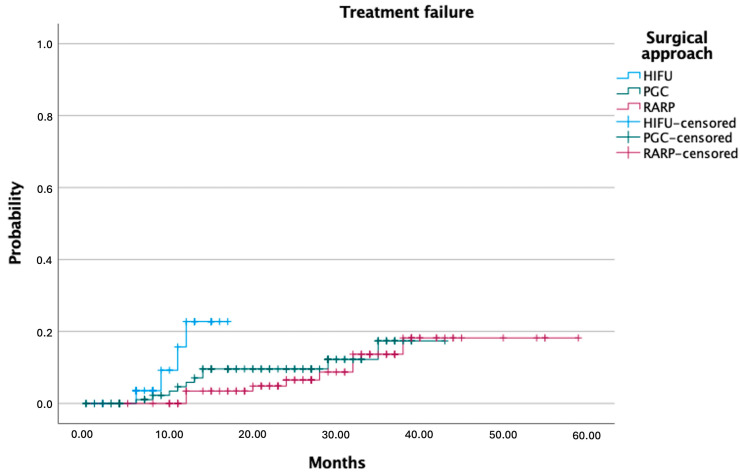
Kaplan–Meier curves showing treatment failure following HIFU, PGC, and RARP.

Median follow-up was 28 months (IQR 24–38) in the RARP group, 23 months (IQR 11–30) in the PGC group, and 8 months (IQR 4–14) for patients treated with HIFU (*p* < 0.001 as shown in Table 3). Time to treatment failure was significantly longer for RARP (median of 24 months with an IQR of 12–36), compared to 20 months (IQR of 8–29) for PGC and 8 months (IQR of 4–13) for HIFU (*p* < 0.001). When applying a liberal definition of failure—including rising PSA requiring rebiopsy or any salvage treatment—overall failure rates were similar across groups (12.8% in RARP, 8.7% in PGC, and 8.1% in HIFU with each *p* > 0.9). The distribution of in-field versus out-of-field recurrences, and the rate of systemic treatment initiation at 24 months, did not differ significantly among cohorts (each *p* > 0.6). Among focal therapy patients experiencing recurrence, 73.5% of failures were anatomically classified as in-field, i.e., within the previously ablated region, while 26.5% occurred out-of-field, in untreated portions of the prostate. This classification was derived from post-treatment imaging correlation (mpMRI and/or PSMA- or choline-based PET/CT) and spatial mapping of all histologically confirmed recurrence sites. In all recurrence cases included in the analysis, histologic confirmation by targeted biopsy was obtained prior to initiating salvage therapy or assigning failure category. The distribution of recurrence types by treatment modality is detailed in Table 3. Kaplan–Meier analysis demonstrated a significantly higher probability of remaining treatment failure–free over time in RARP patients, followed by PGC and then HIFU (Figure 1). Notably, HIFU showed an earlier decline in treatment durability (*p* = 0.01).

### 3.4. Functional Outcomes

**Figure 2:**
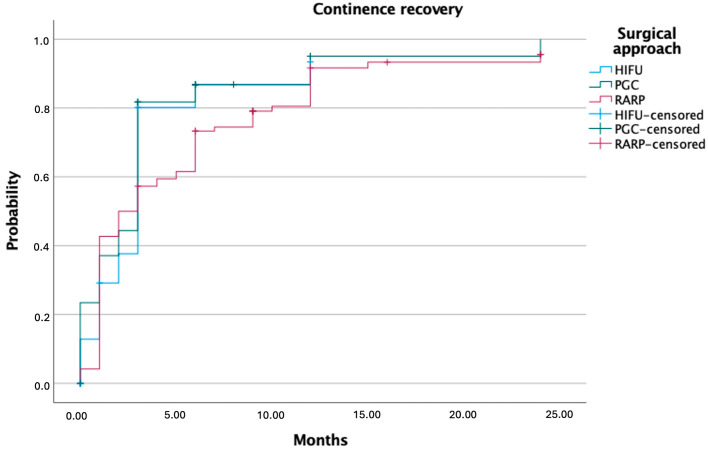
Kaplan–Meier curves showing continence recovery following HIFU, PGC, and RARP.

**Figure 3:**
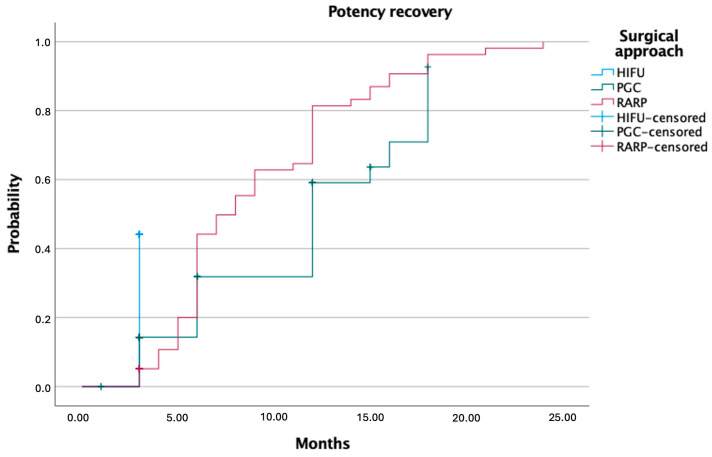
Kaplan–Meier curves showing potency recovery following HIFU, PGC, and RARP.

At last follow-up, urinary continence rates were high and comparable across treatment groups: 89.1% for HIFU, 88.6% for PGC, and 87.8% for RARP (*p* = 0.53 in Table 4). Erectile function recovery differed significantly: 57.7% of patients in the RARP group regained potency, compared to 44.1% after HIFU and 37.5% following PGC (*p* = 0.01). Among patients treated with RARP, 26.6% reported spontaneous erections, 26.6% required phosphodiesterase type 5 (PDE5) inhibitors, and 4.5% used PGE-1. In the HIFU group, 36.8% reported spontaneous erections, while in the PGC group the rate was 19.2%. Kaplan–Meier curves showed that, although erectile function returned earlier in HIFU patients, overall recovery probability remained higher in the RARP group over time (Figure 2 and Figure 3).

## 4. Discussion

The emergence of focal therapies for organ-confined prostate cancer represents a deliberate shift toward treatment individualization, aiming to preserve functional integrity without compromising oncologic control [13,14,15,16,17,18]. While RARP remains the benchmark for whole-gland management, HIFU and PGC have gained increasing attention for patients seeking organ-sparing approaches or deemed unsuitable for surgery [19,20,21,22,23,24,25]. These minimally invasive strategies are not merely technological refinements—they respond to a clinical imperative: mitigating postoperative morbidity while maintaining therapeutic efficacy [26,27,28]. Within this evolving landscape, the present study offers a prospective, single-center comparison of RARP, HIFU, and PGC in patients with localized, low- to intermediate-risk prostate cancer. To our knowledge, this is the first institutional series to evaluate these three modalities head-to-head, using standardized definitions and outcome metrics across perioperative, oncologic, and functional domains. The strength of this analysis lies in its methodological consistency and internal comparability, despite the acknowledged lack of randomization. Differences in baseline characteristics among treatment groups reflect real-world clinical practice, where age, comorbidities, and baseline erectile function often guide therapeutic choice. These differences are not trivial, as they condition treatment selection based on priorities such as oncologic safety, functional preservation, or individual preference. As a result, direct cross-modality comparisons must be interpreted with caution, recognizing that observed differences may stem as much from initial candidacy as from treatment effect. It is equally important to consider that not all individuals diagnosed with low- or favorable intermediate-risk prostate cancer require immediate active treatment. Contemporary guidelines consistently endorse active surveillance as a first-line option for many such patients. Nevertheless, the decision to pursue intervention often arises from a combination of patient anxiety, imaging features (e.g., PIRADS ≥ 3 lesions), histopathologic uncertainty, and multidisciplinary clinical judgment. These dynamics mirror real-world decision-making and justify, in part, the inclusion of patients whose disease might otherwise have been eligible for conservative management. From a perioperative standpoint, RARP was associated with longer operative times but shorter catheterization and hospitalization. In contrast, PGC yielded the highest rate of perioperative complications, primarily due to catheter-related morbidity. This disproportionate burden—particularly urinary retention and orchitis—highlights the importance of appropriate patient counselling when focal treatments are considered, especially in outpatient or fast-track settings. Moreover, extended catheterization in focal therapy patients—typically lasting up to 15 days in cryoablation and approximately one week in most HIFU cases—is often dictated by the extent of treatment. Bilateral ablations or multifocal targeting across both lobes increase the degree of intraprostatic trauma, warranting longer indwelling times to reduce the risk of post-ablative sequelae such as tissue sloughing or incomplete re-epithelialization. While cryoablation aims to prevent ice-slush-related complications through prolonged drainage, HIFU protocols may also require extended catheterization in anatomically complex scenarios. These regimens, although protective in intent, may predispose the patient to a spectrum of catheter-associated adverse events, including irritative symptoms, orchitis, persistent urinary retention, and delayed urethral strictures. Oncologic outcomes favored RARP, which demonstrated longer time to treatment failure and superior failure-free survival on Kaplan–Meier analysis. This advantage likely reflects the extirpative nature of radical surgery, offering complete removal of the gland and periprostatic tissue. Focal modalities, while appealing for their lower invasiveness, inherently target only select regions of the prostate. Even with rigorous imaging and planning, residual disease or marginal misses remain a concern, particularly in the context of multifocal cancer. This was especially evident in the HIFU cohort, where earlier recurrence patterns emerged despite initial parity in early outcomes. In this regard, the spatial distribution of focal therapy failures offers additional granularity: 73.5% of recurrences were classified as in-field, occurring within the prior ablation zone, and likely reflecting limitations in treatment depth or precision. Conversely, 26.5% were deemed out-of-field, involving contralateral or previously untreated regions, potentially linked to occult lesions or subclinical disease undetectable at baseline. This mapping underscores the inherent tension between therapeutic selectivity and oncologic completeness in focal approaches. Functional outcomes were largely preserved across all groups. Urinary continence rates were high regardless of treatment, while erectile function recovery showed a more nuanced trend. Patients undergoing HIFU experienced a faster return to potency during early follow-up, likely due to better preservation of neurovascular integrity. RARP, on the other hand, achieved the highest long-term potency rates. These findings must be interpreted in the context of baseline erectile function, which was lower in the focal therapy cohorts and thus may partially confound comparative recovery profiles. Several limitations must be acknowledged. The lack of randomization and absence of propensity score matching limit the ability to control for selection bias. Although focal procedures were performed by a single high-volume surgeon within a standardized framework, RARP was carried out by multiple operators, potentially introducing variability. Moreover, follow-up duration differed across arms, with shorter surveillance in the HIFU group—a factor that may underestimate the true recurrence rate in this subgroup. The study also lacks a comparator arm including patients managed with radiotherapy or active surveillance, limiting broader generalizability, particularly in low-risk settings where conservative management is common. Finally, the absence of integrated imaging–pathology correlation prevents definitive conclusions regarding anatomical causes of focal treatment failure, such as undetected multifocality or targeting inaccuracies, despite the use of multiparametric MRI and image-guided planning. Despite these constraints, the present findings offer meaningful insights into the clinical trade-offs between extirpative and focal strategies in the management of localized prostate cancer. By harmonizing outcome measures across different therapeutic pathways, this study supports the feasibility of individualized treatment planning rooted in real-world practice. Future research should aim to refine patient selection algorithms, adopt standardized failure criteria, and incorporate radiologic–pathologic integration to strengthen the predictive accuracy and durability of focal approaches. Ultimately, optimal treatment cannot be defined in absolute terms; rather, it should reflect a nuanced balance between oncologic control and preservation of functional quality of life, tailored to each patient’s clinical and personal priorities.

## 5. Conclusions

In this prospective, single-center comparison of RARP, HIFU, and PGC, all three modalities demonstrated high rates of urinary continence preservation and acceptable functional outcomes. However, focal therapies—particularly PGC—were associated with higher catheter-related morbidity and an earlier onset of treatment failure. While HIFU offered faster recovery of erectile function, RARP provided superior oncologic durability over time. These findings underscore the need for individualized treatment selection based on clinical priorities, patient characteristics, and shared decision-making within a standardized comparative framework.

## Figures and Tables

**Table 1:** Baseline characteristics of patients who underwent HIFU, PGC, and RARP.

Variable	HIFU *n* = 49	Cryo *n* = 114	RARP *n *= 109	*p*
**Age (years, median, IQR)**	74 (67.5–75)	74 (70–76)	63 (59–67)	<0.001
**Body Mass Index (Kg/m^2^, median, IQR)**	25 (23–28)	26.7 (24.1–29)	25.8 (23.8–28.7)	0.103
**ASA score (n,%)**				<0.001
**1–2**	40 (81.6%)	65 (57%)	99 (90.8%)	
**3–4**	9 (18.4%)	49 (43%)	10 (9.2%)	
**Preoperative PSA (ng/mL, median, IQR)**	7 (4.8–9.8)	7.5 (5.13–9.9)	6.65 (5.14–8.97)	0.350
**Preoperative IIEF-5 (media, IQR)**	13.5 (4.5–21)	11 (3–19)	21 (18–22)	<0.001
**Prostate Volume (cm^3^, median, IQR)**	44 (30.6–65.2)	50.5 (32–63.7)	41.6 (27–70)	0.133
**Total Biopsy Cores (** ***n*** **, median, IQR)**	15 (10–20)	16 (12–20)	14 (11–16)	0.54
**Total Systematic Biopsies (** ***n*** **, median, IQR)**	15 (10–20)	16 (12–20)	14 (11–16)	0.53
**Total Targeted Biopsies (** ***n*** **, median, IQR)**	3 (2–3)	3 (2–3)	3 (2–3)	0.89
**Total Positive Cores (** ***n*** **, median, IQR)**	4 (2–5)	4 (2–6)	4 (2–6)	0.581
**% Positive Cores (** ***n*** **, median, IQR)**	26.3 (17.6–40)	25.6 (14.6–42.8)	30 (20–50)	0.316
**Year of Procedure (n,%)**				-
**2019**	-	-	29 (26.7%)	
**2020**	-	-	34 (31.1%)	
**2021**	-	31 (27.1%)	46 (42.2%)	
**2022**	-	44 (38.6%)	-	
**2023**	20 (40.8%)	20 (17.6%)	-	
**2024**	29 (59.1%)	19 (16.7%)	-	
**PI-RADS Score (n,%)**				0.251
**2**	3 (6.1%)	9 (7.8%)	5 (4.5%)	
**3**	13 (26.5%)	14 (12.2%)	22 (20.2%)	
**4**	27 (55.2%)	70 (61.5%)	62 (56.9%)	
**5**	6 (12.2%)	21 (18.5%)	20 (18.4%)	
**PI-RADS Area Size (mm, median, IQR)**	10 (7.5–14)	12 (8–15)	10 (8–12.5)	0.226
**Biopsy Gleason Grade Group (n,%)**				0.103
**PGG1**	25 (51%)	41 (36%)	52 (47.7%)	
**PGG2**	24 (49%)	73 (64%)	57 (52.3%)	
**PGG3**	-			
**PGG4**	-			
**cT Stage (n,%)**				<0.001
**1**	49 (100%)	114 (100%)	8 (7.3%)	
**2a**	-		27 (24.8%)	
**2b**	-		25 (23%)	
**2c**	-		49 (44.9%)	
**3a**	-		-	
**Approach (n,%)**				<0.001
**Emigland**	32 (65.3%)	64 (56.1%)	-	
**Whole-Gland**	17 (34.7%)	50 (43.9%)	-	

**Table 2:** Perioperative outcomes.

Variable	HIFU *n* = 49	Cryo *n* = 114	RARP *n * = 109	*p*
**Operative time (minutes, median, IQR)**	45 (35–62)	55 (40–65)	120 (106–141)	<0.001
**Nerve-sparing (intent, n,%)**				-
**Intrafascial (=bilateral)**	-	-	39 (35.8%)	
**Interfascial (=unilateral)**	-	-	59 (54.1%)	
**Extrafascial (=no nerve-sparing)**	-	-	11 (10.1%)	
**pT stage (n,%)**				
**any pT2**			95 (87.1%)	
**pT3a**			14 (12.9%)	
**Surgical margins status (n, type,%)**				-
**Focal**	-	-	7 (6.5%)	
**Positive**	-	-	16 (14.7%)	
**Negative**	-	-	86 (78.8%)	
**Length of hospital stay (days, median, IQR)**	3 (3–3)	2 (2–3)	2 (2–2)	<0.001
**Catheterization time (days, median, IQR)**				
**Time to catheter removal (days, median, IQR)**	7 (7–7)	15 (15–15)	7 (7–9)	<0.001
**Any complication**	10 (20.4%)	36 (31.5%)	3 (2.75%)	<0.001
**Total number of complications**				<0.001
**1st complication**	9 (18.3%)	31 (27.1%)	3 (2.75%)	
**2nd complication**	1 (2.1%)	5 (4.4%)	-	
**Time to 2nd complication (months, median, IQR)**	1 (1–1)	2 (1–5)	-	0.432
**Severe complications (** ***n*** **,%)**	1 (2.1%)	1 (0.87%)	-	0.455
**Clavien Dindo classification (n, detail, %)**				0.04
**1–2**	9 (5 Orchitis, 1 Fever, 3 Hematuria)	(5 Hematuria, 17 Orchitis, 8, Fever, 5 Acute urinary retention	3 (Fever)	
**3–5**	1 (Recto-vesical fistula)	1 (Urethral fistula)	-	

**Table 3:** Oncologic outcomes.

Variable	HIFU *n* = 49	Cryo *n* = 114	RARP *n* = 109	*p*
**Follow-up months (months, median, IQR)**	8 (4–14)	23 (11–30)	28 (24–38)	<0.001
**Neoadjuvant ADT therapy (** ***n*** **,%)**	3 (6.1%)	5 (4.38%)	-	0.078
**Time to treatment failure (months, median, IQR)**	8 (4–13)	20 (8–29)	24 (12–36)	<0.001
**Type of treatment failure (** ***n*** **,%)**				0.99
**In-field recurrence/Biochemical recurrence/**	4 (8.1%)	7 (6.1%)	14 (12.8%)	
**Out-of-field recurrence**	-	3 (2.6%)	-	
**Treatment failure: liberal definition (=rebiopsy for rising PSA, adjuvant radiotherapy, PSA > 0.1)**				0.98
**Treatment failure at last follow-up (** ***n*** **,%)**	4 (8.1%)	10 (8.7%)	14 (12.8%)	
**Need for systemic treatment (n,%)**				0.618
**Systemic treatment at 24 months**	1 (2%)	5 (2.6%)	3 (2.75%)	
**Adjuvant ADT (** ***n*** **,%)**	-	2 (1.75%)	3 (2.75%)	0.679
**Salvage therapy (** ***n*** **,%)**				0.98
**Repeat PGC/HIFU**	2 (4%)	4 (3.5%)	-	
**Radical Prostatectomy**	-	-	-	
**Radiation therapy**	-	-	8 (7.3%)	
**Radiation therapy and ADT**	-	-	2 (1.8%)	
**ADT**	2 (4%)	3 (2.6%)	1 (0.9%)	
**Metastatic disease (** ***n*** **,%)**				-
**24 months**	-	3 (2.63%)	-	

**Table 4:** Functional outcomes.

Variable	HIFU *n* = 49	Cryo *n* = 114	RARP *n* = 109	*p*
**Continence at follow-up %**	89.1%	88.6%	87.8%	0.53
**Sexual recovery at follow-up (details%)**	44.1%	37.5%	57.7%	0.01
**Potency without drugs/device**	18 (36.8%)	22 (19.2%)	29 (26.6%)	
**With PDE5-I**	4 (8.1%)	21 (18.4%)	29 (26.6%)	
**With PGE-1**	-	-	5 (4.5%)	
**No erections**	27 (55.1%)	71 (62.2%)	46 (42.3%)	

## Data Availability

The data presented in this study are available in this article.

## Abbreviations

The following abbreviations are used in this manuscript:

HIFU	High-Intensity Focused Ultrasound
PGC	Prostate Gland Cryoablation
RARP	Robot-Assisted Radical Prostatectomy
GG	Grade Group
mpMRI	Multiparametric Magnetic Resonance Imaging
PSMA	Prostate Specific Membrane Antigen
PET-CT	Positron Emission Tomography-Computed Tomography
BMI	Body Mass Index
ASA	American Society of Anesthesiologists
IIEF	International Index of Erectile Function
PIRADS	Prostate Imaging Reporting and Data System
IQR	Interquartile Range
PDE5	Phosphodiesterase Type 5
PGE-1	Prostaglandin E1
